# Selection on vocal output affects laryngeal morphology in rats

**DOI:** 10.1111/joa.13366

**Published:** 2021-01-21

**Authors:** Raffaela Lesch, Thomas Schwaha, Andrea Orozco, Margaret Shilling, Susan Brunelli, Myron Hofer, Daniel L. Bowling, Betty Zimmerberg, William Tecumseh Fitch

**Affiliations:** ^1^ Department of Behavioral and Cognitive Biology University of Vienna Vienna Austria; ^2^ Department of Evolutionary Biology, Integrative Zoology University of Vienna Vienna Austria; ^3^ Department of Psychology Williams College Williamstown MA USA; ^4^ Department of Psychiatry Columbia University New York NY USA; ^5^ Department of Psychiatry and Behavioral Sciences Stanford University School of Medicine Stanford CA USA

**Keywords:** development, larynx, morphology, rodent communication, selection, ultrasonic vocalizations, vocal anatomy

## Abstract

Although laryngeal morphology often reflects adaptations for vocalization, the structural consequences of selection for particular aspects of vocal behavior remain poorly understood. In this study, we investigated the effects of increased ultrasonic calling in pups on the adult larynx morphology in selectively bred rat lines. Laryngeal morphology was assessed using multiple techniques: mineralized cartilage volumes were compared in 3D‐models derived from microCT scans, internal structure was compared using clearing and staining procedures combined with microscopy, cellular structure was compared using histology and microscopy, and element composition was assessed with scanning energy dispersive X‐ray spectroscopy. Our results show that adult rats from lines bred to produce ultrasonic calls at higher rates as pups have shorter vocal folds and a more mineralized thyroid cartilage compared to rats bred to produce ultrasonic calls at lower rates. The change in vocal fold length appears to account for differences in low‐frequency calls in these two rat lines. We suggest that the observed increases in mineralization of the thyroid cartilage in the high‐ultrasound lineage provide increased reinforcement of the laryngeal structure during ultrasonic call production. Our findings therefore demonstrate an effect of selection for vocal behavior on laryngeal morphology, with acoustic consequences.

## Graphical abstract

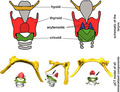

## 1 INTRODUCTION

The mammalian larynx has dual functions of airway protection and acoustic signal production (Shiba, 2009). Satisfying both has produced an organ that is effective for vocal production, but not particularly energetically efficient (Titze, 2018). The functional morphology of the mammalian larynx is relatively well understood, mostly due to extensive research on its role in the production of human speech, and the realization that many of the essential principles (e.g., as described by source‐filter theory and myoelastic‐aerodynamic theory) are widely conserved across mammalian clades (Fitch, 2016; Riede & Fitch, 1999; Titze, 1994). In contrast, the avian syrinx, which functions more purely in sound production due to its location deep in the chest, exhibits a much higher degree of diversity across species (Fitch & Hauser, 2006). Thus, the fact that the larynx must satisfy requirements for at least two critical functions can be estimated to have constrained its evolution and development, particularly with respect to modifications made solely for the purpose of vocal communication.

The mammalian larynx consists of a cartilaginous thyroid, arytenoids, cricoid, epiglottis, and a supporting set of hyoid bones (Harrison, 1995; Schneider, 1964). Despite a high degree of interspecific conservation, a number of impressive modifications of the mammalian larynx have been documented (Fitch, 2016). Among such adaptations we find enlargements of the larynx itself in howler monkeys, pads on the vocal folds in lions, or the ability to produce high frequency whistles in rodents (Dent & Fay, 2018; Dunn et al., 2015; Klemuk et al., 2011). In some rodents, such as rats and mice, another structure, the alar cartilage is added to the “standard” structures of the mammalian larynx (Inagi, Schultz & Ford, 1998).

Murine rodents have two distinct vocalization ranges, defined by low‐frequency calls and ultrasonic vocalizations (USVs) (Brudzynski, 2005, 2018). This provides a very interesting and unusual opportunity to analyze functional changes to larynx morphology. USVs in rats can further be categorized into two distinct ultrasonic vocalization ranges used to communicate different affective states of the caller: 22 kHz vocalizations communicate negative affect whereas 50 kHz calls communicate positive affect (Portfors, 2007). These two ranges are specific to adult rats; when isolated, rat pups produce USVs in a single range centered around 40 kHz (Portfors, 2007). Apart from these well‐studied ultrasonic vocalizations, rats also produce low‐frequency calls within the human hearing range (Brudzynski, 2010, 2018). Extrapolating from what is known about vocalization in other mammals, the production mechanism of low‐frequency calls in rodents is predictable on the basis of “ordinary” laryngeal vocal fold vibration (Herbst et al., 2012; Riede et al., 2011; Roberts, 1975b). Previous studies agree that USVs are also produced in the larynx, but by a different “whistle” mechanism (Johnson et al., 2010; Mahrt et al., 2016; Riede, Borgard & Pasch, 2017; Sanders et al., 2001). However, debate remains over the nature of this mechanism: Mahrt and colleagues suggest a planar impinging jet. In contrast, Riede and colleagues argue for an edge‐tone whistle mechanism (Mahrt et al., 2016; Riede, Borgard & Pasch, 2017). These two different mechanisms are associated with different types of morphological changes in the larynx, for example in the alar cartilage (thought to be essential in the production of USVs in rat communication (Inagi, Schultz & Ford, 1998; Riede, Borgard & Pasch, 2017). This leads to the question addressed by our study: how does selection for USV production rate influence laryngeal anatomy, including both USV‐related anatomy, and morphological characteristics associated with low‐frequency vocal production?

We address this question here by analyzing vocal anatomy in two rat lines that have been selected for over 50 generations to produce low or high rates of USVs as pups, during a maternal separation paradigm (Brunelli et al., 1997; Brunelli & Hofer, 2007). This sustained artificial selection upon USV call rate in pups yielded two distinct rat lines with both different vocal behavior and distinctive stress responses (Brunelli & Hofer, 2007; Zimmerberg, Brunelli & Hofer, 1994). An earlier study of rats from these two selected lines revealed clear effects of selection on vocal acoustics (Lesch et al., 2020), but the morphological basis of the line‐specific acoustic differences documented there remain unknown.

In particular, our previous study showed that selection on rat pup USV rate did not affect acoustic parameters of adult USVs, but rather affected acoustic aspects of adult low‐frequency calls. Specifically, we found that “high line” individuals (i.e., pups selected for increased USV production) were heavier in body weight than “low line” rats (i.e., pups selected for decreased USV production), but did not produce lower frequencies in their calls. This apparent exception to a general correlation (allometry) between body mass and fundamental frequency of low‐frequency calls led us to formulate the hypothesis that selection for acoustic output in pups will lead to changes in adult laryngeal morphology. In the present study, we used multiple visualization and measurement techniques to quantitatively demonstrate an effect of artificial selection for a specific aspect of vocal behavior on the laryngeal morphology of rats. We found that larynges of high line adults have shorter vocal folds and a more mineralized thyroid cartilage compared to low line adults. These results support our hypothesis that selection on pup calling will lead to differential changes in adult laryngeal morphology, depending on rat line.

## 2 METHODS

### 2.1 Rat lines

The rat lines studied here were initially described in Brunelli et al., (1997) and have been bred since the 1990s (57‐58 generations) on the basis of differences in the rate of USV calls produced by pups in response to a maternal separation paradigm. Both lines were developed from the same founding population of N:NIH *Rattus norvegicus domestica*. The N:NIH strain was developed in the 1980s to provide an outbred and heterogeneous strain from eight inbred strains (including the Wistar lineage; Hansen & Spuhler, 1984). From a founding population of these N:NIH rats one line was bred to produce high rates of pup USV calls during a maternal separation paradigm (“high line”), while the “low line” was bred to produce few calls during the same paradigm. In the maternal separation paradigm, the dam was separated from the litter 20 minutes prior to testing, to elicit separation induced calls from the pups. During a two minute test phase each pup's USV call rate was measured. This rate was used as the basis from which the high‐ and low‐lines were bred. Within five generations the high line pups produced up to 300 USVs within two minutes whereas the low line pups produced less than 50 calls. In the 58th generation high line individuals produced on average over 300 calls with some individuals reaching over 400 USVs; low line individuals produced less than 50 USVs with the exception of one individual that produced 86 USVs (Lesch et al., 2020). The breeding was conducted at Williams College Animal Facility and all procedures were approved by the Williams College Animal Care and Use Committee.

### 2.2 Larynx extraction

We dissected larynges from 51 adult rats (between 133 and 135 days old; high line: 14♂, 13♀; low line: 12♂, 12♀) that were culled from the breeding population of the two rat lines maintained at Betty Zimmerberg's laboratory at Williams College in Williamstown, MA, USA. Each larynx was extracted with the hyoid, tongue, and 0.5 cm of trachea still attached. After dissection, the larynges were stored in individual plastic bags and frozen at −25°C. These larynges were double bagged, placed in a cardboard box, surrounded by ice packs, and shipped in an insulated cooler to W. Tecumseh Fitch's lab at the University of Vienna in Vienna, AT. Upon arrival all larynges remained frozen, and were transferred to −21° C freezers.

### 2.3 Overview

Out of the 51 larynges we randomly chose 8 larynges of each group (high male, high female, low male, low female), adding up to a total of 32 larynges. All 32 larynges were used in the microCT analysis and after the scan four of these larynges were stained, three were used for the energy dispersive X‐ray (EDX) and one underwent sectioning.

### 2.4 microCT scans

Our primary method for comparing larynges between lines was based on the volumes of key cartilages. Each laryngeal specimen was scanned using microCT (µCT) to obtain a three‐dimensional computer model that allowed us to quantitatively determine the volume of mineralized tissue in the hyoid bone, and each of the main laryngeal cartilages (cricoid, arytenoids and thyroid).

#### 2.4.1 Specimen preparation

Thirty‐two larynges (8 per sex and line combination) were prepared for microCT scanning at the University of Vienna. The individual larynges were thawed and further dissected for the scanning procedure. The majority of the tongue was cut off and other surplus tissues were removed. We flushed the larynx with 0.9% saline solution to remove any tissue or fluid from within the larynx and trachea. Afterward, we placed the larynx in a plastic tube, stabilized its position with plastic straws and added a drop of saline at the bottom to prevent dessication. The tubes were sealed with parafilm and mounted on the scanner platform for scanning.

#### 2.4.2 Scanning process and visualization

We scanned all larynges with an XRadia MicroXCT‐400 (Carl Zeiss X‐ray Microscopy, Pleasanton, CA, USA) using the 0.4x detector assembly. Scanning parameters were set to a source voltage of 40 kVp and 200 µA beam intensity. Projection images were recorded with 1 s exposure time (detector binning = 4) per projection and an angular increment of 0.250° between projections. Tomographic sections were reconstructed using the XMReconstructor software supplied with the scanner. Isotropic voxel size in the reconstructed volumes was 45.1 μm. Reconstructed volumes were exported in Digital Imaging and Communications in Medicine (DICOM) format.

DICOM image stacks were loaded in the Amira software package (version 6.4.0) and the following structures were segmented manually: thyroid, arytenoids, cricoid, tracheal rings, and the hyoid apparatus. Since the microCT scans only reliably capture mineralized structures, these reconstructions only show the mineralized parts of the cartilages and hyoid apparatus. We used the threshold tool in the Amira segmentation editor to aid manual segmentation of the cartilages and hyoid apparatus (basihyal, ceratohyal, chondrohyal, hypohyal and thyrohyal; Sharma & Sivaram, 1967). The threshold tool was adjusted for each individual to perfectly capture the hyoid apparatus and these same settings were then used for the rest of the (mineralized) larynx. We measured the mineralized volume of the hyoid, cricoid, arytenoids and thyroid and vocal fold length on the reconstructed 3D models of all larynges. Vocal fold length was approximated based on the mineralization patterns of the 3D reconstructions. We placed landmarks on the dorsal border of each arytenoid cartilage, and in the area of vocal fold attachment on the thyroid cartilage; we then measured the distance between the arytenoid and thyroid landmarks, resulting in two vocal fold measurements per individual (right and left; Bowling et al., 2020). We recorded the average value as “mean vocal fold length” for each individual to be used for all further statistical analysis.

### 2.5 Clearing and staining

To visualize the entire (and not just mineralized) laryngeal anatomy in our specimens, we selected four larynges (high male: #18, high female: #42, low male: #34, low female: #2; microCT scans and 3D reconstructions were done for all specimens) to create cleared and stained specimens, according to an adapted staining protocol based on Rigueur and Lyons (Rigueur & Lyons, 2014). Excess tissue was mechanically removed for faster preparation. The main steps in the preparation were fixation of frozen larynges in 4% formaldehyde, followed by the first staining step in an ethanolic Alcian blue solution for staining cartilage. Afterwards, soft tissue was preincubated and macerated in potassium hydroxide (KOH) solution, followed by staining in aqueous Alizarin Red solution in KOH. Additional soft tissue removal and clearing was then accomplished using KOH. Whole specimens were finally transferred into glycerol for documentation and analysis with a Nikon SMZ25 stereomicroscope equipped with a Nikon DsRi‐2 camera (Nikon Instruments, Tokyo, Japan) or a Hirox RH‐2000 digital microscope system (Hirox, Limonest, France).

### 2.6 Electron microscopy and energy dispersive X‐ray spectroscopy (EDX)

To assess the elemental composition of the mineralization we used three larynges (high line male #19, low line male #26, high line female #15) for elemental analysis. The distribution of elements was identified using EDX throughout cartilages of the larynx and the hyoid bone. Both the cricoid and thyroid cartilage were cut in half and attached to carbon adhesive discs on aluminum stubs with their cut surfaces facing upward. The hyoid bones were attached to the carbon disks as a whole. Additional conductive carbon cement (Leit‐C) was applied to enable better contact to the stubs and prevent excessive charging. Prior to analysis, specimens were carbon coated with a Leica EM MED20 (Leica Microsystems, Wetzlar, Germany). Analysis was conducted with a Jeol‐IT3000 scanning electron microscope with the following parameters: BED‐C, 20 kV, WD 11 mm, STd.P.C 63.7‐67.7, 100 Pa and 350‐600 times magnification.

### 2.7 Histological analysis

To distinguish between calcification and ossification of the mineralized parts, one larynx (high line male #25) was embedded in epoxy resin for sectioning and histological analysis. The specimen was first fixed as described in the clearing and staining section. It was then decalcified with 20% EDTA and dehydrated with acidified dimethoxypropane prior to embedding into Agar LVR resin (Agar Scientific, Stansted, UK) using acetone as an intermediate. Sections of cured resin blocks were sliced at 1 µm section thickness with a Leica UC6 ultramicrotome equipped with a diamond knife (Diatome, Nidau, Switzerland) and analyzed with a Nikon NiU compound microscope.

### 2.8 Statistical analysis

To determine the effect of line breeding on vocal fold length and mineralized cartilage volumes we constructed separate generalized linear models. The models compare each of the following measurements between lines: cricoid mineralized volume, thyroid mineralized volume, arytenoid mineralized volume and vocal fold length. A Gamma log distribution was used for all models. The significance of line specific differences was assessed by comparing the predictive value of full models against null models lacking the relevant predictor. In the full models we included the covariate bodyweight and a dummy coded variable representing line and the respective sex within line. For all model comparisons, the null model only included the covariate ‘body weight’ due to our default allometric expectation that larger individuals should have larger vocal structures, and accounting for the fact that the different selected rat lines showed significant differences in body weight. The covariate bodyweight was scaled and centered before running any analysis. All models were inspected and plotted to determine if model assumptions were satisfied. All variance inflation factors were <4 and overdispersion was <0.4.

Statistical analyses were performed in R (Version 3.6.1; www.r‐project.org/) and R‐Studio (Version 1.2.1335; www.rstudio.com/) using the following packages and versions: performance_0.4.4, boot_1.3‐22, plyr_1.8.4, readxl_1.3.1, openxlsx_4.1.0.1, forcats_0.4.0, MuMIn_1.43.6, ggthemes_4.2.0, car_3.0‐3, carData_3.0‐2, MASS_7.3‐51.4, lme4_1.1‐21, Matrix_1.2‐17, stringr_1.4.0, dplyr_0.8.3, purrr_0.3.2, readr_1.3.1, tidyr_0.8.3, tibble_2.1.3, ggplot2_3.2.0, and NCmisc_1.1.6.

## 3 RESULTS

### 3.1 Cartilage volumes, vocal fold lengths, and bodyweight allometry

**FIGURE 1:**
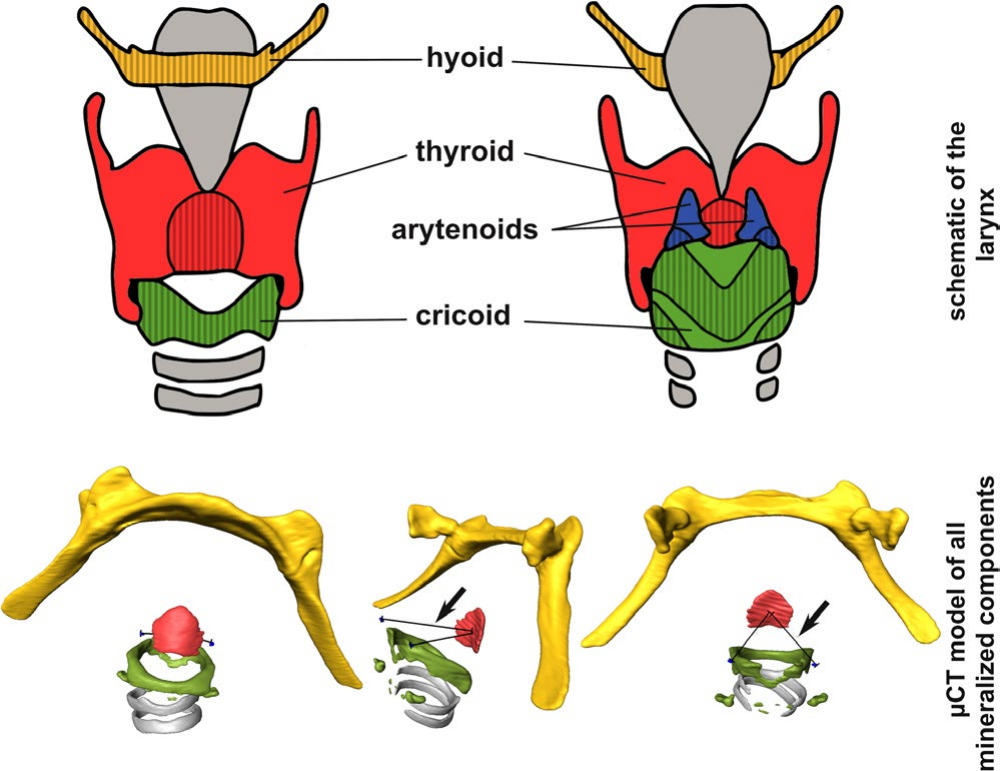
Schematic drawing of a mammalian larynx and 3D reconstructions of the mineralized larynx components of a high line male rat (#H16). The hyoid is figured in yellow, the thyroid in red, the arytenoids in blue and the cricoid in green. The trachea and epiglottis are shown in grey. The vertical‐line shading in the schematic drawing indicates the mineralization visible in the microCT scans. An alar cartilage is not represented in the schematic and was not visible in our microCT scans. The epiglottis also was not visible in the scans. Vocal fold length measurements are indicated in the 3D reconstructions with thin black lines between the thyroid and arytenoid cartilages and pointed out by black arrows

**FIGURE 2:**
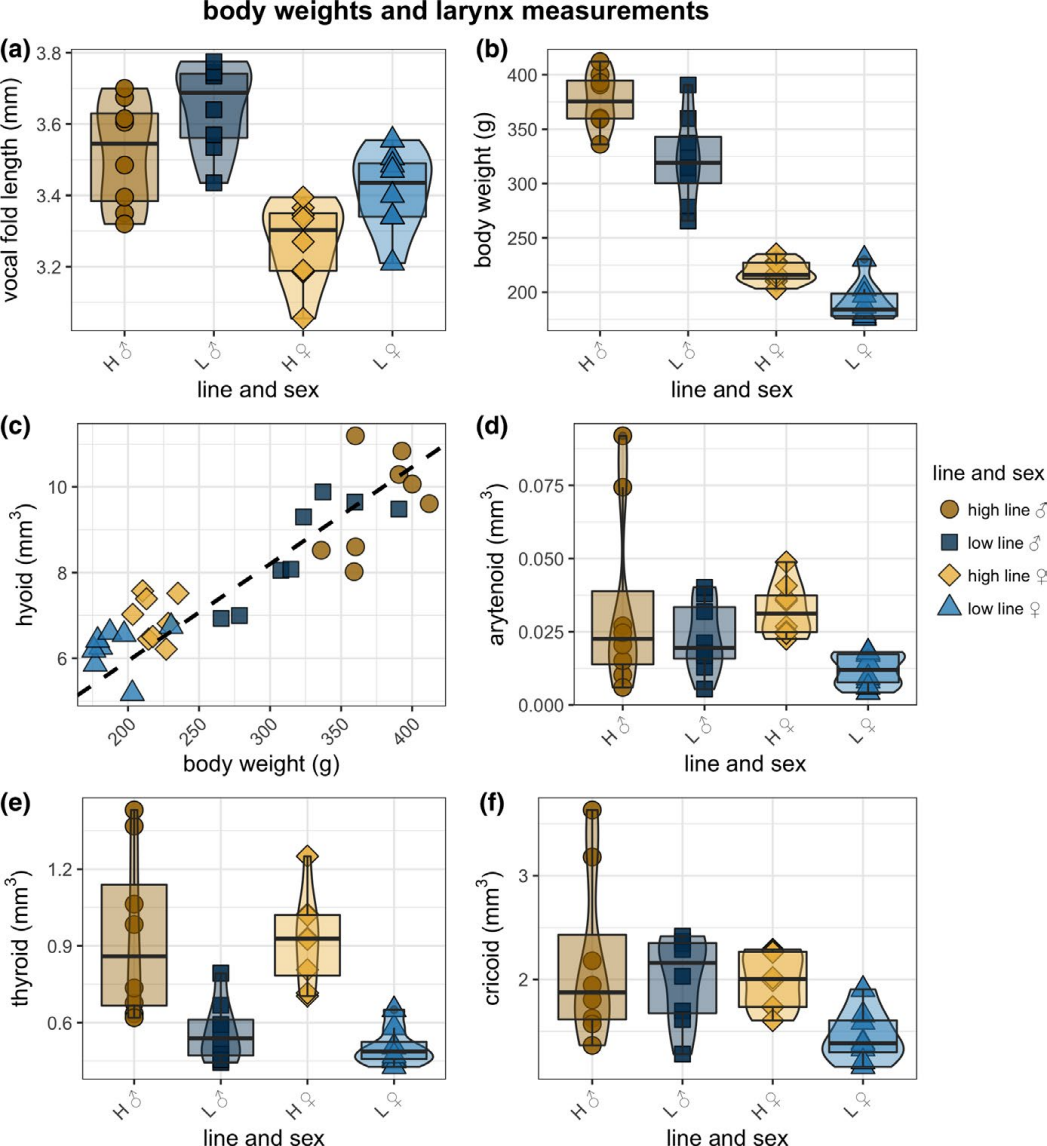
Overview of bodyweight, vocal fold length and volume of mineralized parts of the cartilages and the hyoid bone for the two selected lines, by sex. The different lines are indicated by letters (H = high line, L = low line), and sex is indicated by symbols (♂ = male, ♀ = female). Circles indicate high line males, squares indicate low line males, diamonds indicate high line females and triangles indicate low line females. A: Mean vocal fold length of each individual in mm plotted in groups of line and sex. B: Body weight in grams of all individuals plotted in groups of line and sex. C: Reconstructed hyoid volume of all 32 specimen plotted relative to their body weight. D‐F: Volumes of specific laryngeal cartilages for all 32 CT specimens, plotted in groups of line and sex

The reconstructed larynx models allowed us to measure a proxy of vocal fold length and quantify the mineralized volumes of each cartilage and the hyoid apparatus (Figure 1). The full models for vocal fold length as well as arytenoid, thyroid, and cricoid volume were all significantly superior to the null models (Tables 1, 2, 3, 4); only the full model for the hyoid was not significantly better than the null model (Table 5). Therefore differences between lines were identified for all major laryngeal cartilages, independent of body size, suggesting deviations for expected allometry (Figure 2d‐f). The hyoid is the only vocal structure whose volume follows the expected size allometry: larger animals have a larger hyoid bone and therefore a greater mineralized volume (Figure 2c). The three measured cartilages deviate from this simple bodyweight‐volume allometry indicating that selection on the different rat lines significantly affects the degree of mineralization in laryngeal cartilages. (Tables 1, 2, 3, 4).

**TABLE 1:** Null/full model comparison and summary of the model for vocal fold length. High line males are included in the intercept

Vocal fold length	
	Pr(>Chi)
full/null model comparison	5.38 * 10^−6^

	estimate	std. error	t value	Pr(>|t|)
intercept^a^	1.18827	0.02541	46.755	<2 * 10^−16^
bodyweight g	0.05633	0.01844	3.054	0.00503
low line male	0.07383	0.02031	3.636	0.00115
high line female	0.03663	0.03954	0.926	0.36252
low line female	0.09932	0.04533	2.191	0.03727

^a^ Intercept includes high line males

**TABLE 2:** Null/full model comparison and summary of the model for cricoid volume. High line males are included in the intercept

Cricoid volume	
	Pr(>Chi)
full/null model comparison	0.002512

	estimate	std. error	t value	Pr(>|t|)
intercept^a^	1.0912	0.1807	6.04	1.9 * 10^−6^
low line male	−0.2339	0.1443	−1.62	0.11678
high line female	−0.605	0.2811	−2.152	0.04048
low line female	−1.0136	0.3222	−3.145	0.00401
bodyweight g	−0.2749	0.1311	−2.097	0.04549

^a^ Intercept includes high line males

**TABLE 3:** Null/full model comparison and summary of the model for arytenoid volume. High line males are included in the intercept

Arytenoid volume	
	Pr(>Chi)
full/null model comparison	0.002138

	estimate	std. error	t value	Pr(>|t|)
intercept^a^	−2.8381	0.4713	−6.022	2 * 10^−6^
low line male	−0.6067	0.3766	−1.611	0.118
high line female	−0.9778	0.7333	−1.333	0.1935
low line female	−2.1325	0.8407	−2.537	0.0173
bodyweight g	−0.5337	0.342	−1.56	0.1303

^a^ Intercept includes high line males

**TABLE 4:** Null/full model comparison and summary of the model for thyroid volume. High line males are included in the intercept

Thyroid volume	
	Pr(>Chi)
full/null model comparison	6.1 * 10^−9^

	estimate	std. error	t value	Pr(>|t|)
intercept^a^	0.07738	0.18877	0.41	0.685101
low line male	−0.57998	0.15082	−3.846	0.000665
high line female	−0.24695	0.2937	−0.841	0.40783
low line female	−0.88356	0.33671	−2.624	0.01412
bodyweight g	−0.12008	0.13699	−0.877	0.388478

^a^ Intercept includes high line males

**TABLE 5:** Null/full model comparison and summary of the model for hyoid volume. Since the full model was not significantly better than the null model analysis was stopped here

Hyoid volume	
	Pr(>Chi)
full/null model comparison	0.7564

**FIGURE 3:**
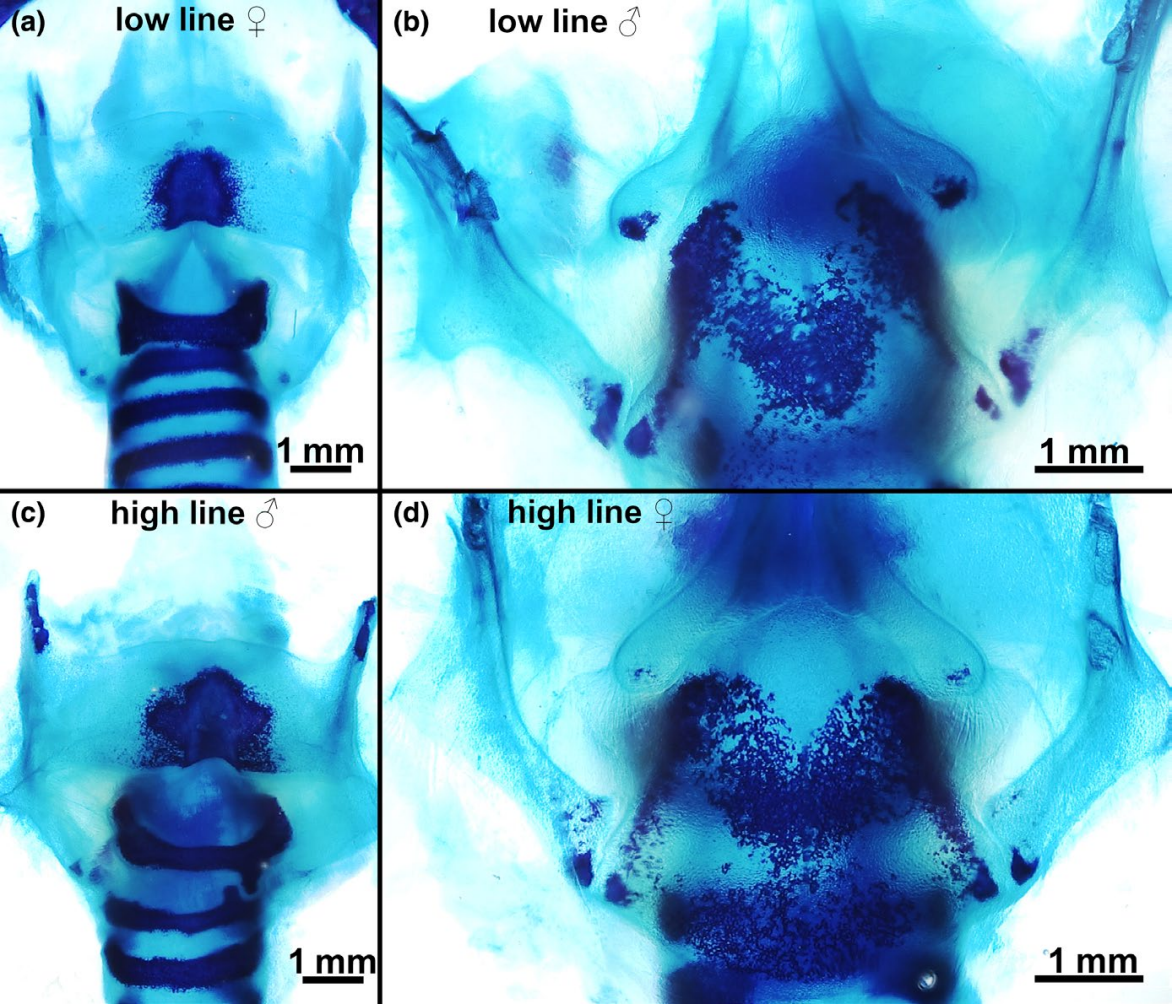
Selection of photographs of the 4 specimens whole mount stains. Calcified material is stained in purple and cartilage in blue. The high line individual shows a much more calcified thyroid compared to the low line. A: Ventral view of the larynx and hyoid of a low line female (#2). B: Dorsal view of the cricoid and arytenoids of a low line male (#34). C: Ventral view a larynx (lacking the hyoid) of a high line male (#18). D: Dorsal view of the thyroid and cricoid of a high line female (#42)

The mineralized portions of the cartilages were as follows (see Figure 1): In the thyroid a shield‐like portion centered on the insertion points of the vocal folds was mineralized; the arytenoids were mineralized on the dorsal transitions to the cricoid; the cricoid ring itself and the dorsal “shield” were mineralized in the shape of an “M” from the arytenoids to the center of the cartilage (e.g. Figure 1, right panel; Figure 3d). For the thyroid cartilage, the mineralization difference is clear with high line males and females having a more mineralized thyroid than the low line (Figure 2e). In the arytenoids and cricoid the difference in mineralization is less clear and may just be caused by one group (low line females; Figure 2d,f).

A deviation from simple bodyweight allometry also applies to vocal fold length (Figure 2a); although high line individuals are on average larger in size, they have *shorter* vocal folds compared to the smaller low line individuals, which have *longer* vocal folds (Figure 2b). Thus, selection for vocalization during infancy had clear and statistically significant effects on adult larynx morphology, and led to a deviation from simple allometric predictions.

### 3.2 Composition and nature of mineralized areas

**FIGURE 4:**
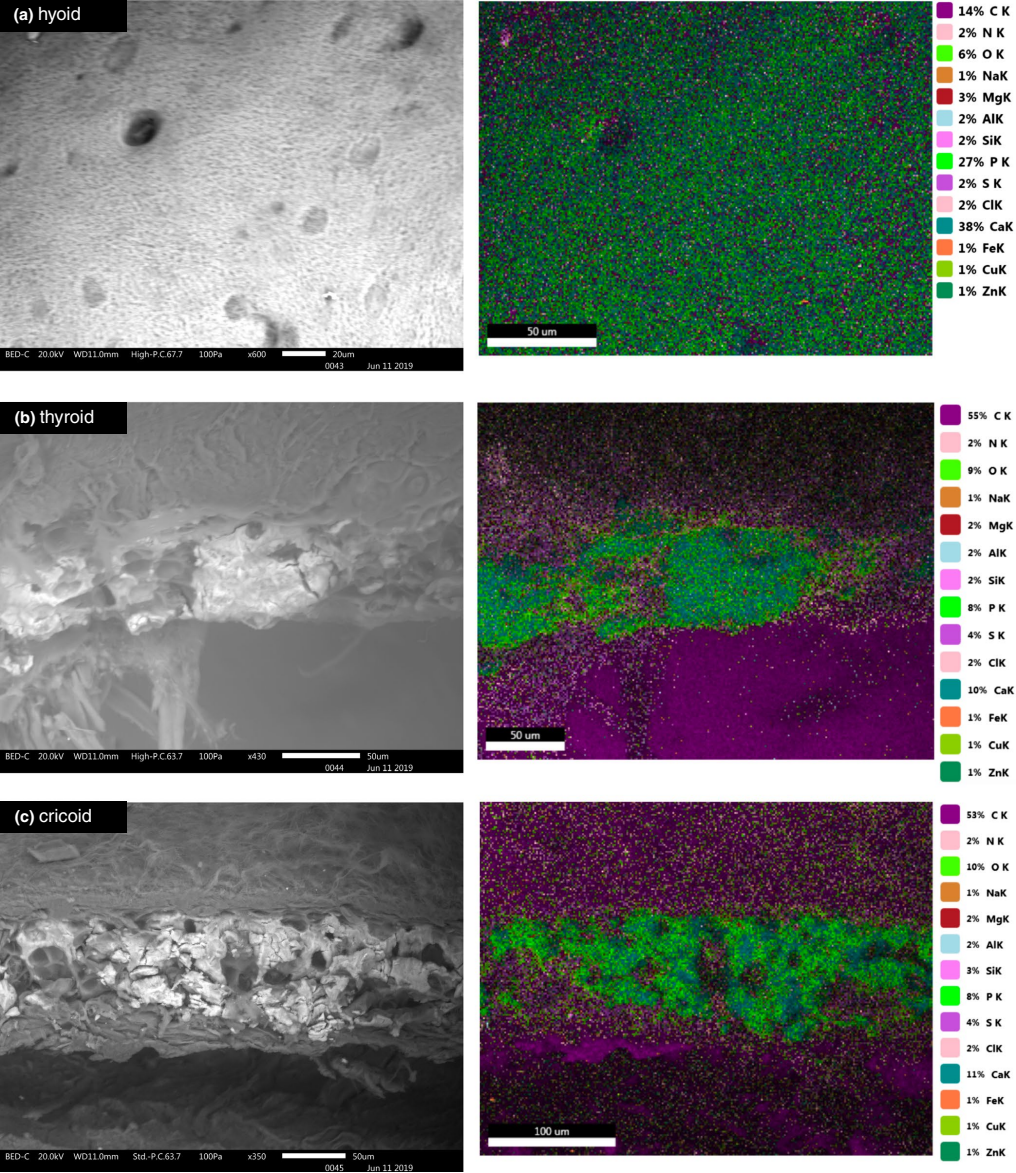
Photographs and elemental analysis (element map) from one individual (#19, high line male) showing the element distribution in the cricoid and thyroid in comparison to the hyoid. The cross sections of the mineralized regions in the laryngeal cartilages show a similar composition to the hyoid. A: Photograph and element map of the hyoid surface. B: Photograph and element map of the cross section of the thyroid. C: Photograph and element map of a cross section of the cricoid

**FIGURE 5:**
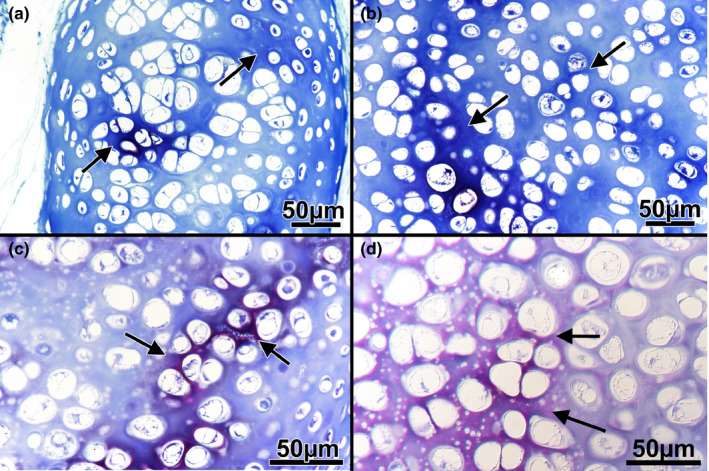
Cross‐sections of a mineralized thyroid cartilage of a high line male (#25). Semi‐thin sections, stained with toluidine blue. Mineralized areas are shown by intense staining indicated by arrows in all images. A, B: Overview of two sections showing hyaline cartilage with several chondrons. C, D: Details of mineralized areas located mainly in the inter‐territorial matrix between chondrons

Clearing and staining of larynges was used to determine the nature of mineralized structures; with our staining methods calcified structures are stained purple and cartilage stains blue (Figure 3). These specimens confirmed that the mineralized cartilage volumes indicated in microCT scans are calcifications (Figure 1). Additional elemental analysis using EDX of the cross sections of the mineralized regions in laryngeal cartilages show the same elemental components as the hyoid bone (Figure 4a‐c). Both show a high degree of phosphate and calcium which is typical for hydroxyapatite (the mineralization of bone and calcified cartilage). Histological sections of the cartilage, including mineralized areas, show typical hyaline cartilage with chondrocytes clustering to form chondrons in a large structural matrix (Figure 5a,b). Mineralized areas stain more intensely than the remaining cartilaginous matrix (Figure 5) and located in various patches of the territorial and interterritorial matrix between chondrons. This distribution around chondrocytes indicates that the mineralized areas represent calcified cartilage rather than true bone tissue.

## 4 DISCUSSION

We investigated the impact of selective breeding for increased or decreased infant USVs on adult rat larynx morphology, finding that adult rats from lines bred to produce more USVs during maternal separation as pups had substantially more calcified thyroid cartilages, and shorter vocal folds. The opposite was true for rats bred to produce few USVs during maternal separation as pups, which had longer vocal folds and less‐calcified thyroid cartilages as adults. This confirms our hypothesis that selective breeding can lead to changes in laryngeal morphology. Our anatomical findings are consistent with previous evaluations of acoustic measures in the same rat lines (Lesch et al., 2020).

USVs are at the center of research associated with emotional states, and are often used as an easily measured proxy for stress response styles in rats (Branchi, Santucci & Alleva, 2001; Brunelli, 2005; Simola & Brudzynski, 2018). Therefore it is important to document and understand possible changes to the vocal production mechanisms that may arise as a result of selective breeding (and, by extension, natural selection). In Lesch et al. (2020), analyses of vocal outputs from rat lines selectively‐bred for USVs as pups showed that line breeding affected the fundamental frequency of low‐frequency calls but not USVs in adults. The likely mechanism by which these low‐frequency calls are produced is standard vocal fold vibration in the larynx, as detailed in the myo‐elastic aerodynamic theory (Elemans et al., 2015; Titze, 2006), implicating potential differences in vocal fold length as the main cause of these differing fundamental frequencies (Herbst et al., 2012; Riede et al., 2011; Roberts, 1975a). Our approximate measurements of vocal fold length from microCT reconstructions showed that high line individuals indeed have shorter vocal folds, which is consistent with the higher fundamental frequency of their low‐frequency calls. In contrast, low line individuals have longer vocal folds and a correspondingly lower fundamental frequency. The differences in vocal fold length found in the current study thus explain the line difference we observed in the fundamental frequency of low‐frequency calls in our previous study (i.e. high line males producing higher frequencies than low line males; Lesch et al., 2020).

Furthermore, our microCT reconstructions revealed significant differences in mineralization of the thyroid cartilage in the two rat lines. While the significant difference in mineralization was clearly line specific in the thyroid cartilage, the significant differences in mineralization in the arytenoids and cricoid might just be driven by females of the low line. Histological analysis of stained larynges suggested that the mineralized components observed in the reconstructed microCT scans are indeed calcifications. Further EDX analyses confirmed that these calcifications in the laryngeal cartilages have the elemental components of hydroxyapatite [Ca_5_(PO_4_)_3_(OH)], present in both bone and calcified cartilage (Boskey, 1988). The histological structure, however, shows patchy distribution of the mineralized areas between chondrocytes, indicating calcified cartilage rather than bone. Definitive distinction of calcified cartilage vs bone would require specific staining against collagen I for bone and collagen II for cartilage (Fratzl, 2008; Hall, 2015).

Based on a comprehensive review of mammalian laryngeal anatomy, Schneider (1964) concluded that there is no generalizable rule for the process of calcification in the mammalian larynx, and suggested that the thyroid cartilage in eutherians tends to first calcify in areas that are under less deformation pressure, for example, inferior and superior thyroid horn (Schneider, 1964). This prediction does not match the pattern of calcification we found in our rat larynges, which were mainly calcified in areas that can be under significant deformation pressure, specifically the specific areas of vocal fold attachment. Carter (2020) compared calcifications in the trachea and larynx across laryngeally echolocating bats and found that higher‐intensity vocalizing species tend to have a more mineralized thyroid cartilages than lower‐intensity species. Thyroid mineralization in the high line rats might help to facilitate and sustain USV production, similar to that observed in laryngeally echolocating bats. However, it remains unclear whether this mineralization is genetically determined irrespective of vocalization, or whether increased rates of vocalization in young animals might have led to the increased mineralization of thyroid cartilages that we observe in adults. Detailed anatomical investigations through development, for example, using microCT, would be necessary to test this prediction.

A limitation of this study is the fact that we cannot determine whether the line specific differences that we have documented would develop reliably based purely on genetics, whether they result from behavioral differences (e.g., high vocalization rates) having a developmental effect on morphology, or a combination of the two. More precisely, the observed changes to laryngeal morphology might be caused by differences in usage during early developmental stages and/or genetically determined developmental differences. For example, low line individuals might have a less calcified thyroid simply due to lower rates of USV production (“use it or lose it”); while high line individuals, due to their frequent vocalizations, provide more frequent stress that provides the stimulus for increased mineralization. Addressing this question would require detailed individual‐specific lifelong documentation of vocalization rates, and/or molecular‐genetic and developmental investigations. However, this question of developmental causation is secondary to the primary questions addressed here, that is, does selection on vocalization affect laryngeal morphology, and if so how.

Another limitation is that, because this is the first investigation of laryngeal anatomy in these rat lines, we cannot determine when the anatomical changes we have documented have occurred during the 50+ generations of ongoing selection. Behaviorally, changes in pup vocalization occurred quite rapidly, in the first five generations of selection (Brunelli et al., 1997). But this does not necessarily indicate that the anatomical changes occurred equally rapidly. Unfortunately, addressing this question would entail starting the selection experiment afresh from unselected lines, and acquiring anatomical specimens at each generation, a research program far beyond the scope of our study.

It is well known that, despite its relatively conservative structure, the form of the mammalian larynx can be modified to suit its particular functions (Charlton & Reby, 2016; Fitch, 2016). For example, the larynx and hyoid of howler monkeys are enormously enlarged to support their extremely loud, low‐frequency vocalizations, and these changes reflect specific selection in different species of howler monkeys (Dunn et al., 2015). The large “roaring cats” (lions, tigers, jaguars, and leopards) have several modifications of vocal fold structure and hyoid morphology that again support the production of loud, low‐frequency vocalizations (Klemuk et al., 2011; Titze et al., 2010). Many similar modifications for loud low‐frequency vocalizations have been documented in a range of ungulates (Frey et al., 2007; Frey et al., 2011; Frey & Hofmann, 2000; Frey & Riede, 2003). Nonhuman primates show a suite of specific vocal modifications, including many types and sizes of laryngeal air sac (Fitch, 2016; Schön Ybarra, 1995), and large‐scale comparisons of laryngeal morphology in primates and carnivores suggest that the primate larynx has evolved more rapidly than that of carnivores (Bowling et al., 2020).

Given these well‐known modifications on an evolutionary time‐scale, it is clearly important to better understand how selection for specific vocal traits (whether lower‐ or higher‐frequency calls, louder calls, or more frequent calling) affects the morphology and development of the main sound producing organ, the larynx (that is, to examine both the ontogeny and phylogeny of changes in vocal anatomy). Our study takes a first step in this direction and reveals that two specific aspects of laryngeal structure ‐ vocal fold length and thyroid mineralization – can be modified by artifical selection on rate of vocalization alone, and in a relatively short time span. Furthermore, it should be noted that this targeted selection produced a host of other “side‐effects,” including, for example, on voice fundamental frequency, body size, and temperament. This implies that selection on the voice can have a considerable diversity of effects that are of clear relevance for an animal's behavior and fitness.

Finally, our results also show that the expected allometric correlation between overall body size and the size of specific organs can easily be overridden by selection, at least with respsect to laryngeal size. Although our high line rats were larger in overall body size, they had shorter vocal folds and produced vocalizations with higher fundamental frequencies. Thus, our results are consistent with the hypothesis that laryngeal morphology, and the vocal acoustic parameters that depend on it such as fundamental frequency, is relatively unconstrained by overall body size in mammals (Fitch & Hauser, 2006; Garcia et al., 2017). Put differently, under strong selection for vocal output, laryngeal size can change independent of body size. This offers comparative insight into the peculiar fact that human males, due to laryngeal hypertrophy, have a much lower fundamental frequency than women (about 50%) despite being on average only 20% larger (Pisanski et al., 2014), and belies the common assumption that fundamental frequency automatically reflects body size as “a law of physics” (Morton, 1977). The presence or absence of acoustic allometry – the scaling of bioacoustic parameters with body size (e.g., Bowling et al., 2017) – additionally depends strongly on biological factors, specifically whether laryngeal morphology correlates with overall body size. This is a matter of anatomy and physiology, not of physics.

To sum up, our data support the hypothesis that rats selectively bred for USV production as pups show changes in larynx morphology in adulthood, which in turn have clear and predictable results on vocal production. This finding is clearly relevant to researchers using USVs to gain insight into the emotional state and stress response, as well as to broader questions in the evolution of communication (cf. Bowling et al., 2020; Dunn et al., 2015; Fitch, 2016).

## CONFLICT OF INTEREST

The authors state no conflict of interest.

## AUTHOR CONTRIBUTIONS

R.L., D.B., B.Z., and W.T.F contributed to conceptualization. R.L. contributed to data curation and project administration. R.L., D.B., and T.S. contributed to formal analysis. W.T.F., B.Z., and M.H. contributed to funding acquisition. R.L., T.S., B.Z., A.O., and M.S. contributed to investigation. R.L., W.T.F, and T.S. contributed to methodology. T.S., W.T.F., B.Z., M.H., and S.B. contributed to resources. R.L., T.S., and D.B. contributed to software. W.T.F. contributed to supervision. R.L. and T.S. contributed to validation and visualization. R.L., W.T.F., and T.S. contributed to writing—first draft. R.L., T.S., D.B., W.T.F., and B.Z contributed to writing—review and editing.

### Open Research Badges

This article has earned an Open Data badge for making publicly available the digitally‐shareable data necessary to reproduce the reported results. The data is available at http://doi.org/10.5281/zenodo.4415263.

## Supporting information

Supplementary Material

Supplementary Material

Supplementary Material
